# Tricarbonylbis­(triphenyl­phosphane-κ*P*)iridium(I) hexa­fluoridophosphate methanol monosolvate

**DOI:** 10.1107/S1600536812035593

**Published:** 2012-08-23

**Authors:** Ilana Engelbrecht, Hendrik G. Visser, Andreas Roodt

**Affiliations:** a Department of Chemistry, University of the Free State, PO Box 339, Bloemfontein, 9300, South Africa

## Abstract

In the title compound, [Ir(C_18_H_15_P)_2_(CO)_3_]PF_6_·CH_3_OH, the Ir^I^ atom is coordinated by two triphenyl­phosphine ligands in axial sites and three carbonyl ligands in the equatorial plane of a fairly regular trigonal bipyramid: the equatorial C—Ir—C angles range from 115.45 (9) to 126.42 (10)°. The small deviations from the ideal tetra­hedral geometry around the P atoms are illustrated by C—P—C angles ranging from 104.08 (9) to 106.46 (9)°. In the crystal, the mol­ecules are linked by weak C—H⋯F, C—H⋯O and C—H⋯π inter­actions.

## Related literature

For related complexes, see: Randall *et al.* (1991 ▶, 1994 ▶); Raper & McDonald (1973 ▶). For other *P*-donor ligands, see: Purcell *et al.* (1995 ▶); Otto & Roodt (2001 ▶); Otto *et al.* (2005 ▶); Muller *et al.* (2008 ▶). For their use in catalytic olefin transformation reactions, see: Haumann *et al.* (2004 ▶); Crous *et al.* (2005 ▶); Booyens *et al.* (2007 ▶); Ferreira *et al.* (2007 ▶).
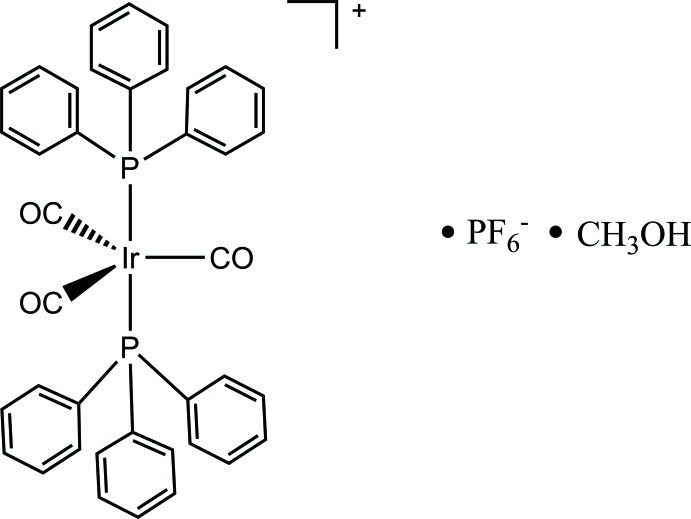


## Experimental

### 

#### Crystal data

[Ir(C_18_H_15_P)_2_(CO)_3_]PF_6_·CH_4_O
*M* *_r_* = 977.80
Monoclinic, 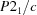

*a* = 16.487 (5) Å
*b* = 13.571 (4) Å
*c* = 20.903 (5) Å
β = 125.297 (5)°
*V* = 3817 (2) Å^3^
*Z* = 4
Mo *K*α radiation
μ = 3.69 mm^−1^
*T* = 100 K
0.18 × 0.14 × 0.06 mm

#### Data collection

Bruker APEXII CCD diffractometer
Absorption correction: multi-scan (*SADABS*; Bruker, 2008 ▶) *T*_min_ = 0.556, *T*_max_ = 0.809
68611 measured reflections
9501 independent reflections
8699 reflections with *I* > 2σ(*I*)
*R*_int_ = 0.037

#### Refinement

*R*[*F* ^2^ > 2σ(*F* ^2^)] = 0.019
*wR*(*F* ^2^) = 0.044
*S* = 1.03
9501 reflections
489 parameters
H-atom parameters constrained
Δρ_max_ = 1.01 e Å^−3^
Δρ_min_ = −0.75 e Å^−3^

### 

Data collection: *APEX2* (Bruker, 2011 ▶); cell refinement: *SAINT-Plus* (Bruker, 2008 ▶); data reduction: *SAINT-Plus*; program(s) used to solve structure: *SHELXS97* (Sheldrick, 2008 ▶); program(s) used to refine structure: *SHELXL97* (Sheldrick, 2008 ▶); molecular graphics: *DIAMOND* (Brandenburg & Putz, 2005 ▶); software used to prepare material for publication: *WinGX* (Farrugia, 1999 ▶).

## Supplementary Material

Crystal structure: contains datablock(s) global, I. DOI: 10.1107/S1600536812035593/hb6932sup1.cif

Structure factors: contains datablock(s) I. DOI: 10.1107/S1600536812035593/hb6932Isup2.hkl

Additional supplementary materials:  crystallographic information; 3D view; checkCIF report

## Figures and Tables

**Table 1:** Hydrogen-bond geometry (Å, °) *Cg*1, *Cg*2 and *Cg*3 are the centroids of the C11–C16, C21–C26 and C41–C46 rings, respectively.

*D*—H⋯*A*	*D*—H	H⋯*A*	*D*⋯*A*	*D*—H⋯*A*
C15—H15⋯F3^i^	0.95	2.39	3.281 (3)	157
C16—H16⋯F6^i^	0.95	2.53	3.319 (3)	141
C42—H42⋯F6^i^	0.95	2.38	3.138 (3)	136
C43—H43⋯F2^i^	0.95	2.49	3.386 (3)	158
C45—H45⋯O01^ii^	0.95	2.50	3.281 (3)	139
C64—H64⋯F4^iii^	0.95	2.47	3.200 (3)	133
O01—H01⋯F3^i^	0.84	2.27	3.059 (3)	157
C53—H53⋯*Cg*1^iv^	0.95	2.68	3.523 (2)	148
C35—H35⋯*Cg*3^iv^	0.95	2.91	3.587 (2)	129
C13—H13⋯*Cg*2^v^	0.95	2.97	3.744 (2)	140

Symmetry codes: (i) 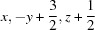; (ii) 
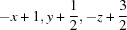
; (iii) 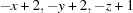; (iv) 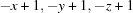; (v) 
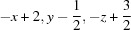
.

**Fig. 1.**
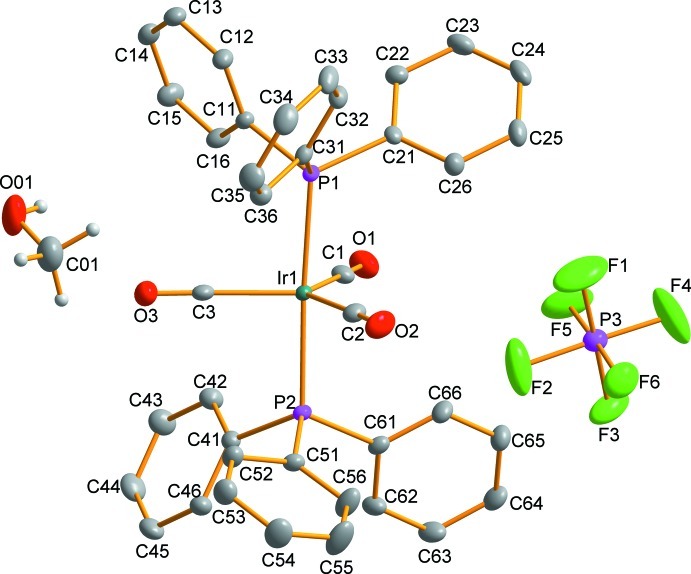
Molecular structure of the title compound. Displacement ellipsoids are drawn at the 50% probability level. Hydrogen atoms have been omitted for clarity.

## supplementary crystallographic information

### Comment

P donor ligands (Muller *et al.*, 2008; Purcell *et al.*, 1995; Otto *et al.*, 2005; Otto & Roodt, 2001) form part of ongoing research in different catalytic olefin transformation reactions such as hydroformylation (Haumann *et al.*, 2004; Crous *et al.*, 2005), metathesis (Booyens *et al.*, 2007) and methoxycarbonylation (Ferreira *et al.*, 2007). As part of our studies in this area, we now describe the structure of the title compound: all bond distances and angles fall within the range for similar complexes (Randall *et al.* 1991, 1994; Raper & McDonald, 1973).

The main fragment of the crystal structure of the title compound, [Ir(CO)_3_(PPh_3_)_2_](PF_6_).MeOH, was originally reported by Randall *et al.*, 1991, in the trigonal form, crystallizing in the space group *R*3 with hydrogen sulfate as counter ion. In this case, the Ir(I) complex (Figure 1) crystallizes with one hexafluoridophosphate anion and a methanol solvent molecule in the *P*2_1_/*c* spacegroup. The trigonal bipyramidal complex consists of three carbonyl groups in the equatorial plane and two triphenylphosphine ligands in the axial plane.

Similar Ir—P distances (2.3620 (8) and 2.3599 (8) Å) and P1—Ir—P2 angle of 177.047 (18) ° make the phosphine ligands equally *trans*. Ir—C3 distance of 1.947 (2) Å is slightly longer than for Ir—C1 and Ir—C2 distances, both equal to 1.938 (2) Å. Ir—C—O angles are close to linear (175.6 (2) - 178.8 (2) °) and C—O distances range from 1.107 (3) - 1.135 (3) Å, with C3—O3 distance the shortest. Angles between the equatorial ligands show some distortion with C2—Ir1—C3 = 115.45 (9) ° and C1—Ir1—C3 = 118.13 (9) ° compared to C2—Ir1—C1 = 126.42 (10) °. C—P—C angles range from 104.07 (9) - 106.46 (9) ° illustrating the distorted tetrahedral geometry around the P atoms.

In the crystal, weak C—H···F, C—H···O and C—H···π, interactions link the molecules into a supra-molecular network (Table 1).

### Experimental

CO was bubbled through a solution of [Ir(COD)(PPh_3_)_2_]PF_6_ (cod = 1,5-cyclooctadiene) (50.0 mg, 0.0515 mmol) in benzene while the mixture was vigorously stirred under gentle reflux. Rapid displacement of COD occurs after which all solvents were evaporated. The product was filtered after the addition of methanol and diethyl ether. Slow evaporation of methanol solution gave yellow blocks. (Yield: 40.1 mg, 82%)

### Refinement

The methine and aromatic H atoms were placed in geometrically idealized positions at C—H = 1.00 and 0.95 Å, respectively and constrained to ride on their parent atoms, with *U*_iso_(H) = 1.2*U*_eq_(C). The highest peak is located 0.79 Å from Ir1 and the deepest hole is situated 0.79 Å from F4.

### Crystal data

**Table:**

[Ir(C_18_H_15_P)_2_(CO)_3_]PF_6_·CH_4_O	*F*(000) = 1928
*M**_r_* = 977.80	*D*_x_ = 1.701 Mg m^−^^3^
Monoclinic, *P*2_1_/*c*	Mo *K*α radiation, λ = 0.71069 Å
Hall symbol: -P 2ybc	Cell parameters from 9817 reflections
*a* = 16.487 (5) Å	θ = 2.6–28.3°
*b* = 13.571 (4) Å	µ = 3.69 mm^−^^1^
*c* = 20.903 (5) Å	*T* = 100 K
β = 125.297 (5)°	Block, yellow
*V* = 3817 (2) Å^3^	0.18 × 0.14 × 0.06 mm
*Z* = 4	

### Data collection

**Table:**

Bruker APEXII CCD diffractometer	8699 reflections with *I* > 2σ(*I*)
Graphite monochromator	*R*_int_ = 0.037
φ and ω scans	θ_max_ = 28.3°, θ_min_ = 1.9°
Absorption correction: multi-scan (*SADABS*; Bruker, 2008)	*h* = −21→19
*T*_min_ = 0.556, *T*_max_ = 0.809	*k* = −18→15
68611 measured reflections	*l* = −27→27
9501 independent reflections	

### Refinement

**Table:**

Refinement on *F*^2^	Primary atom site location: structure-invariant direct methods
Least-squares matrix: full	Secondary atom site location: difference Fourier map
*R*[*F*^2^ > 2σ(*F*^2^)] = 0.019	Hydrogen site location: inferred from neighbouring sites
*wR*(*F*^2^) = 0.044	H-atom parameters constrained
*S* = 1.03	*w* = 1/[σ^2^(*F*_o_^2^) + (0.0153*P*)^2^ + 4.3801*P*] where *P* = (*F*_o_^2^ + 2*F*_c_^2^)/3
9501 reflections	(Δ/σ)_max_ = 0.004
489 parameters	Δρ_max_ = 1.01 e Å^−^^3^
0 restraints	Δρ_min_ = −0.75 e Å^−^^3^

### Special details

**Table:**

Experimental. The intensity data were collected on a Bruker X8 ApexII 4 K Kappa CCD diffractometer using an exposure time of ?? s/frame. A total of ??? frames were collected with a frame width of 0.5\ % covering up to θ = 28.0 ° with 99.9% completeness accomplished.Spectroscopy data: ^1^H NMR (300 MHz, (CD_3_)_2_CO): δ = 7.5–7.8 (m, 30H). ^31^P NMR (121 MHz, (CD_3_)_2_CO): δ = -1.6 (*s*), -143.0 (m, PF_6_). ν(CO): 1989, 2008, 2025 cm^-1^.
Geometry. All s.u.'s (except the s.u. in the dihedral angle between two l.s. planes) are estimated using the full covariance matrix. The cell s.u.'s are taken into account individually in the estimation of s.u.'s in distances, angles and torsion angles; correlations between s.u.'s in cell parameters are only used when they are defined by crystal symmetry. An approximate (isotropic) treatment of cell s.u.'s is used for estimating s.u.'s involving l.s. planes.
Refinement. Refinement of *F*^2^ against ALL reflections. The weighted *R*-factor *wR* and goodness of fit *S* are based on *F*^2^, conventional *R*-factors *R* are based on *F*, with *F* set to zero for negative *F*^2^. The threshold expression of *F*^2^ > 2σ(*F*^2^) is used only for calculating *R*-factors(gt) *etc*. and is not relevant to the choice of reflections for refinement. *R*-factors based on *F*^2^ are statistically about twice as large as those based on *F*, and *R*- factors based on ALL data will be even larger.

### Fractional atomic coordinates and isotropic or equivalent isotropic displacement parameters (Å^2^)

**Table:**

	*x*	*y*	*z*	*U*_iso_*/*U*_eq_	
C1	0.85491 (15)	0.69659 (15)	0.67007 (13)	0.0193 (4)	
C01	0.6389 (2)	0.5239 (2)	0.77777 (18)	0.0440 (7)	
H01A	0.6237	0.5943	0.7739	0.066*	
H01B	0.5781	0.4873	0.74	0.066*	
H01C	0.6881	0.5132	0.7663	0.066*	
C2	0.70334 (17)	0.63622 (15)	0.47318 (13)	0.0203 (4)	
C3	0.64299 (15)	0.57286 (14)	0.59226 (12)	0.0171 (4)	
C11	0.84576 (14)	0.41711 (14)	0.68487 (11)	0.0147 (4)	
C12	0.85577 (16)	0.31492 (15)	0.68521 (12)	0.0183 (4)	
H12	0.8498	0.2832	0.6422	0.022*	
C13	0.87431 (16)	0.25962 (15)	0.74810 (13)	0.0209 (4)	
H13	0.8808	0.1901	0.7479	0.025*	
C14	0.88347 (16)	0.30508 (16)	0.81104 (13)	0.0216 (4)	
H14	0.8963	0.2668	0.854	0.026*	
C15	0.87397 (17)	0.40654 (17)	0.81152 (13)	0.0238 (5)	
H15	0.8806	0.4378	0.855	0.029*	
C16	0.85477 (16)	0.46261 (15)	0.74836 (12)	0.0194 (4)	
H16	0.8478	0.5321	0.7486	0.023*	
C21	0.95198 (14)	0.51021 (14)	0.62908 (12)	0.0143 (4)	
C22	1.03624 (15)	0.46978 (15)	0.69615 (13)	0.0199 (4)	
H22	1.0308	0.4353	0.7331	0.024*	
C23	1.12827 (16)	0.48030 (17)	0.70857 (14)	0.0257 (5)	
H23	1.1859	0.4531	0.7543	0.031*	
C24	1.13651 (16)	0.52994 (16)	0.65505 (14)	0.0239 (5)	
H24	1.1995	0.5358	0.6637	0.029*	
C25	1.05332 (17)	0.57136 (16)	0.58874 (14)	0.0227 (4)	
H25	1.0594	0.6059	0.5522	0.027*	
C26	0.96128 (16)	0.56221 (15)	0.57591 (12)	0.0189 (4)	
H26	0.9044	0.5914	0.5309	0.023*	
C31	0.77070 (15)	0.40644 (14)	0.52184 (11)	0.0153 (4)	
C32	0.82977 (17)	0.34357 (15)	0.51186 (13)	0.0186 (4)	
H32	0.9001	0.348	0.5461	0.022*	
C33	0.78507 (18)	0.27437 (16)	0.45141 (14)	0.0236 (5)	
H33	0.8251	0.2313	0.4448	0.028*	
C34	0.68332 (19)	0.26832 (17)	0.40143 (14)	0.0273 (5)	
H34	0.6533	0.2209	0.3605	0.033*	
C35	0.62365 (18)	0.33144 (17)	0.41046 (13)	0.0261 (5)	
H35	0.5533	0.3274	0.3755	0.031*	
C36	0.66749 (16)	0.39985 (16)	0.47064 (13)	0.0207 (4)	
H36	0.627	0.4425	0.4771	0.025*	
C41	0.60501 (15)	0.81216 (14)	0.60849 (12)	0.0162 (4)	
C42	0.66416 (16)	0.78861 (16)	0.68786 (12)	0.0201 (4)	
H42	0.7243	0.7532	0.7092	0.024*	
C43	0.63557 (17)	0.81664 (18)	0.73602 (13)	0.0254 (5)	
H43	0.6761	0.8006	0.7902	0.03*	
C44	0.54769 (18)	0.86814 (17)	0.70476 (15)	0.0273 (5)	
H44	0.5286	0.8881	0.7379	0.033*	
C45	0.48746 (17)	0.89072 (16)	0.62554 (14)	0.0250 (5)	
H45	0.4267	0.9249	0.6043	0.03*	
C46	0.51604 (16)	0.86333 (15)	0.57727 (13)	0.0206 (4)	
H46	0.4752	0.8793	0.5231	0.025*	
C51	0.54265 (15)	0.78888 (14)	0.44876 (11)	0.0160 (4)	
C52	0.46604 (15)	0.72272 (16)	0.42633 (13)	0.0212 (4)	
H52	0.4704	0.6798	0.4641	0.025*	
C53	0.38338 (17)	0.71896 (17)	0.34916 (13)	0.0253 (5)	
H53	0.3314	0.6735	0.334	0.03*	
C54	0.37697 (18)	0.78165 (19)	0.29442 (13)	0.0298 (5)	
H54	0.3195	0.7806	0.2418	0.036*	
C55	0.4536 (2)	0.8458 (2)	0.31567 (14)	0.0367 (6)	
H55	0.4495	0.8874	0.2774	0.044*	
C56	0.53717 (18)	0.84969 (17)	0.39324 (13)	0.0273 (5)	
H56	0.5899	0.8938	0.4078	0.033*	
C61	0.72295 (15)	0.89687 (14)	0.56249 (11)	0.0160 (4)	
C62	0.69580 (16)	0.98774 (15)	0.57625 (13)	0.0212 (4)	
H62	0.641	0.9916	0.5798	0.025*	
C63	0.74831 (16)	1.07195 (16)	0.58478 (13)	0.0247 (5)	
H63	0.7293	1.1336	0.5938	0.03*	
C64	0.82869 (17)	1.06647 (16)	0.58012 (13)	0.0241 (5)	
H64	0.8649	1.1244	0.5862	0.029*	
C65	0.85644 (17)	0.97694 (16)	0.56662 (13)	0.0232 (5)	
H65	0.9117	0.9733	0.5637	0.028*	
C66	0.80312 (17)	0.89215 (15)	0.55739 (13)	0.0207 (4)	
H66	0.8217	0.8308	0.5475	0.025*	
O1	0.92145 (12)	0.73141 (12)	0.72421 (10)	0.0309 (4)	
O01	0.67752 (15)	0.49056 (17)	0.85393 (12)	0.0471 (5)	
H01	0.7328	0.5175	0.8858	0.071*	
O2	0.67796 (14)	0.63197 (12)	0.40991 (10)	0.0315 (4)	
O3	0.58781 (12)	0.53708 (12)	0.59882 (10)	0.0289 (4)	
F1	0.9118 (2)	0.70324 (13)	0.40752 (13)	0.0768 (7)	
F2	0.80028 (14)	0.79216 (18)	0.41110 (11)	0.0678 (6)	
F3	0.88792 (15)	0.92851 (11)	0.43228 (10)	0.0497 (5)	
F4	0.99908 (15)	0.8406 (2)	0.42799 (15)	0.0823 (8)	
F5	0.95832 (15)	0.79933 (13)	0.51178 (9)	0.0612 (6)	
F6	0.83935 (13)	0.83280 (13)	0.32776 (9)	0.0436 (4)	
P1	0.82838 (4)	0.48994 (3)	0.60498 (3)	0.01235 (9)	
P2	0.65123 (4)	0.78758 (4)	0.54975 (3)	0.01324 (10)	
P3	0.90070 (4)	0.81519 (4)	0.41996 (3)	0.02208 (12)	
Ir1	0.737840 (5)	0.637207 (5)	0.578891 (4)	0.01155 (3)	

### Atomic displacement parameters (Å^2^)

**Table:**

	*U*^11^	*U*^22^	*U*^33^	*U*^12^	*U*^13^	*U*^23^
C1	0.0173 (10)	0.0155 (9)	0.0248 (11)	0.0027 (8)	0.0119 (9)	−0.0008 (8)
C01	0.0380 (16)	0.0500 (17)	0.0486 (17)	−0.0059 (13)	0.0276 (14)	0.0050 (14)
C2	0.0247 (11)	0.0148 (9)	0.0231 (11)	0.0036 (8)	0.0148 (9)	0.0028 (8)
C3	0.0192 (10)	0.0129 (9)	0.0193 (10)	0.0051 (7)	0.0111 (8)	0.0033 (7)
C11	0.0152 (9)	0.0134 (9)	0.0157 (9)	0.0012 (7)	0.0089 (8)	0.0029 (7)
C12	0.0227 (10)	0.0138 (9)	0.0221 (10)	0.0004 (8)	0.0151 (9)	0.0000 (8)
C13	0.0227 (11)	0.0145 (9)	0.0261 (11)	0.0026 (8)	0.0144 (9)	0.0042 (8)
C14	0.0220 (11)	0.0228 (11)	0.0193 (10)	0.0028 (8)	0.0116 (9)	0.0074 (8)
C15	0.0323 (12)	0.0226 (11)	0.0175 (10)	0.0031 (9)	0.0151 (9)	0.0012 (8)
C16	0.0252 (11)	0.0141 (9)	0.0188 (10)	0.0016 (8)	0.0126 (9)	0.0006 (8)
C21	0.0156 (9)	0.0112 (8)	0.0191 (9)	−0.0007 (7)	0.0119 (8)	−0.0032 (7)
C22	0.0193 (10)	0.0158 (9)	0.0231 (10)	0.0014 (8)	0.0113 (9)	0.0012 (8)
C23	0.0162 (10)	0.0230 (11)	0.0312 (12)	0.0028 (8)	0.0097 (9)	−0.0021 (9)
C24	0.0184 (10)	0.0218 (11)	0.0369 (13)	−0.0046 (8)	0.0190 (10)	−0.0099 (9)
C25	0.0256 (11)	0.0220 (10)	0.0297 (11)	−0.0068 (8)	0.0213 (10)	−0.0062 (9)
C26	0.0197 (10)	0.0189 (10)	0.0199 (10)	−0.0020 (8)	0.0124 (9)	−0.0009 (8)
C31	0.0203 (10)	0.0116 (8)	0.0170 (9)	−0.0027 (7)	0.0125 (8)	−0.0007 (7)
C32	0.0229 (11)	0.0163 (9)	0.0223 (10)	−0.0030 (8)	0.0164 (9)	−0.0026 (8)
C33	0.0354 (13)	0.0198 (10)	0.0290 (11)	−0.0067 (9)	0.0264 (11)	−0.0075 (9)
C34	0.0379 (13)	0.0244 (11)	0.0253 (11)	−0.0109 (10)	0.0215 (11)	−0.0102 (9)
C35	0.0236 (11)	0.0272 (11)	0.0225 (11)	−0.0077 (9)	0.0104 (10)	−0.0061 (9)
C36	0.0211 (10)	0.0175 (10)	0.0235 (10)	−0.0013 (8)	0.0129 (9)	−0.0007 (8)
C41	0.0182 (10)	0.0128 (9)	0.0200 (10)	−0.0001 (7)	0.0124 (8)	−0.0003 (7)
C42	0.0201 (10)	0.0214 (10)	0.0183 (10)	0.0020 (8)	0.0107 (9)	−0.0013 (8)
C43	0.0271 (12)	0.0296 (12)	0.0188 (10)	0.0013 (9)	0.0129 (9)	−0.0038 (9)
C44	0.0315 (13)	0.0272 (12)	0.0326 (13)	−0.0019 (9)	0.0240 (11)	−0.0093 (10)
C45	0.0235 (11)	0.0203 (10)	0.0352 (13)	0.0038 (8)	0.0193 (10)	−0.0017 (9)
C46	0.0193 (10)	0.0183 (10)	0.0249 (11)	0.0033 (8)	0.0131 (9)	0.0034 (8)
C51	0.0154 (9)	0.0142 (9)	0.0148 (9)	0.0017 (7)	0.0067 (8)	0.0010 (7)
C52	0.0179 (10)	0.0217 (10)	0.0216 (10)	0.0002 (8)	0.0100 (9)	0.0052 (8)
C53	0.0187 (11)	0.0272 (11)	0.0245 (11)	−0.0036 (9)	0.0094 (9)	0.0008 (9)
C54	0.0238 (12)	0.0374 (13)	0.0170 (10)	−0.0045 (10)	0.0054 (9)	0.0017 (9)
C55	0.0366 (14)	0.0411 (15)	0.0195 (11)	−0.0104 (11)	0.0088 (11)	0.0101 (10)
C56	0.0272 (12)	0.0264 (11)	0.0191 (11)	−0.0097 (9)	0.0081 (10)	0.0021 (9)
C61	0.0175 (10)	0.0123 (9)	0.0144 (9)	−0.0004 (7)	0.0070 (8)	0.0014 (7)
C62	0.0168 (10)	0.0151 (9)	0.0277 (11)	0.0006 (8)	0.0105 (9)	−0.0013 (8)
C63	0.0221 (11)	0.0147 (10)	0.0304 (12)	−0.0002 (8)	0.0111 (10)	−0.0029 (8)
C64	0.0260 (11)	0.0158 (10)	0.0270 (11)	−0.0059 (8)	0.0132 (10)	−0.0004 (8)
C65	0.0271 (11)	0.0198 (10)	0.0287 (11)	−0.0054 (9)	0.0196 (10)	−0.0020 (9)
C66	0.0283 (11)	0.0134 (9)	0.0264 (11)	−0.0018 (8)	0.0191 (10)	−0.0006 (8)
O1	0.0211 (8)	0.0299 (9)	0.0331 (9)	−0.0048 (7)	0.0107 (7)	−0.0090 (7)
O01	0.0427 (12)	0.0584 (14)	0.0467 (12)	−0.0160 (10)	0.0296 (10)	0.0019 (10)
O2	0.0462 (11)	0.0303 (9)	0.0228 (9)	0.0040 (8)	0.0226 (8)	0.0037 (7)
O3	0.0280 (9)	0.0250 (8)	0.0375 (10)	0.0001 (7)	0.0211 (8)	0.0052 (7)
F1	0.136 (2)	0.0262 (9)	0.0671 (13)	0.0119 (11)	0.0580 (14)	−0.0049 (9)
F2	0.0527 (11)	0.1141 (18)	0.0538 (11)	−0.0414 (12)	0.0407 (10)	−0.0299 (12)
F3	0.0909 (14)	0.0237 (8)	0.0456 (10)	0.0033 (8)	0.0458 (10)	0.0005 (7)
F4	0.0368 (11)	0.129 (2)	0.0927 (17)	−0.0237 (12)	0.0442 (12)	−0.0429 (15)
F5	0.0807 (14)	0.0453 (10)	0.0234 (8)	0.0142 (9)	0.0104 (9)	0.0076 (7)
F6	0.0566 (11)	0.0478 (9)	0.0238 (8)	−0.0030 (8)	0.0216 (8)	0.0000 (7)
P1	0.0147 (2)	0.0100 (2)	0.0142 (2)	0.00026 (17)	0.00941 (19)	0.00037 (17)
P2	0.0144 (2)	0.0102 (2)	0.0147 (2)	0.00088 (17)	0.0081 (2)	0.00118 (18)
P3	0.0224 (3)	0.0202 (3)	0.0206 (3)	−0.0001 (2)	0.0107 (2)	−0.0002 (2)
Ir1	0.01286 (4)	0.00918 (4)	0.01346 (4)	0.00053 (3)	0.00809 (3)	0.00089 (3)

### Geometric parameters (Å, º)

**Table:**

Ir1—C1	1.938 (2)	C35—H35	0.95
Ir1—C2	1.938 (2)	C36—H36	0.95
Ir1—C3	1.947 (2)	C41—C42	1.391 (3)
Ir1—P1	2.3620 (8)	C41—C46	1.398 (3)
Ir1—P2	2.3599 (8)	C41—P2	1.811 (2)
C1—O1	1.128 (3)	C42—C43	1.389 (3)
C01—O01	1.405 (3)	C42—H42	0.95
C01—H01A	0.98	C43—C44	1.386 (3)
C01—H01B	0.98	C43—H43	0.95
C01—H01C	0.98	C44—C45	1.386 (3)
C2—O2	1.135 (3)	C44—H44	0.95
C3—O3	1.107 (3)	C45—C46	1.388 (3)
C11—C16	1.392 (3)	C45—H45	0.95
C11—C12	1.396 (3)	C46—H46	0.95
C11—P1	1.815 (2)	C51—C56	1.384 (3)
C12—C13	1.386 (3)	C51—C52	1.392 (3)
C12—H12	0.95	C51—P2	1.815 (2)
C13—C14	1.380 (3)	C52—C53	1.386 (3)
C13—H13	0.95	C52—H52	0.95
C14—C15	1.387 (3)	C53—C54	1.380 (3)
C14—H14	0.95	C53—H53	0.95
C15—C16	1.393 (3)	C54—C55	1.379 (3)
C15—H15	0.95	C54—H54	0.95
C16—H16	0.95	C55—C56	1.396 (3)
C21—C22	1.395 (3)	C55—H55	0.95
C21—C26	1.398 (3)	C56—H56	0.95
C21—P1	1.815 (2)	C61—C66	1.388 (3)
C22—C23	1.391 (3)	C61—C62	1.397 (3)
C22—H22	0.95	C61—P2	1.818 (2)
C23—C24	1.378 (3)	C62—C63	1.382 (3)
C23—H23	0.95	C62—H62	0.95
C24—C25	1.386 (3)	C63—C64	1.386 (3)
C24—H24	0.95	C63—H63	0.95
C25—C26	1.386 (3)	C64—C65	1.384 (3)
C25—H25	0.95	C64—H64	0.95
C26—H26	0.95	C65—C66	1.392 (3)
C31—C36	1.394 (3)	C65—H65	0.95
C31—C32	1.398 (3)	C66—H66	0.95
C31—P1	1.816 (2)	O01—H01	0.84
C32—C33	1.395 (3)	P3—F1	1.5698 (19)
C32—H32	0.95	P3—F2	1.5870 (19)
C33—C34	1.374 (3)	P3—F3	1.5933 (17)
C33—H33	0.95	P3—F4	1.569 (2)
C34—C35	1.397 (3)	P3—F5	1.5890 (17)
C34—H34	0.95	P3—F6	1.5943 (16)
C35—C36	1.384 (3)		
			
O1—C1—Ir1	177.8 (2)	C45—C46—C41	119.9 (2)
O01—C01—H01A	109.5	C45—C46—H46	120
O01—C01—H01B	109.5	C41—C46—H46	120
H01A—C01—H01B	109.5	C56—C51—C52	119.69 (19)
O01—C01—H01C	109.5	C56—C51—P2	121.59 (16)
H01A—C01—H01C	109.5	C52—C51—P2	118.61 (16)
H01B—C01—H01C	109.5	C53—C52—C51	120.4 (2)
O2—C2—Ir1	175.6 (2)	C53—C52—H52	119.8
O3—C3—Ir1	178.8 (2)	C51—C52—H52	119.8
C16—C11—C12	119.26 (18)	C54—C53—C52	119.7 (2)
C16—C11—P1	120.58 (15)	C54—C53—H53	120.2
C12—C11—P1	120.06 (15)	C52—C53—H53	120.2
C13—C12—C11	120.23 (19)	C55—C54—C53	120.3 (2)
C13—C12—H12	119.9	C55—C54—H54	119.8
C11—C12—H12	119.9	C53—C54—H54	119.8
C14—C13—C12	120.3 (2)	C54—C55—C56	120.3 (2)
C14—C13—H13	119.8	C54—C55—H55	119.9
C12—C13—H13	119.8	C56—C55—H55	119.9
C13—C14—C15	120.0 (2)	C51—C56—C55	119.6 (2)
C13—C14—H14	120	C51—C56—H56	120.2
C15—C14—H14	120	C55—C56—H56	120.2
C14—C15—C16	120.0 (2)	C66—C61—C62	119.29 (19)
C14—C15—H15	120	C66—C61—P2	121.26 (16)
C16—C15—H15	120	C62—C61—P2	119.44 (16)
C11—C16—C15	120.14 (19)	C63—C62—C61	120.3 (2)
C11—C16—H16	119.9	C63—C62—H62	119.8
C15—C16—H16	119.9	C61—C62—H62	119.8
C22—C21—C26	119.65 (19)	C62—C63—C64	120.0 (2)
C22—C21—P1	121.71 (16)	C62—C63—H63	120
C26—C21—P1	118.50 (15)	C64—C63—H63	120
C23—C22—C21	119.5 (2)	C65—C64—C63	120.2 (2)
C23—C22—H22	120.2	C65—C64—H64	119.9
C21—C22—H22	120.2	C63—C64—H64	119.9
C24—C23—C22	120.5 (2)	C64—C65—C66	119.8 (2)
C24—C23—H23	119.8	C64—C65—H65	120.1
C22—C23—H23	119.8	C66—C65—H65	120.1
C23—C24—C25	120.4 (2)	C61—C66—C65	120.3 (2)
C23—C24—H24	119.8	C61—C66—H66	119.9
C25—C24—H24	119.8	C65—C66—H66	119.9
C26—C25—C24	119.8 (2)	C01—O01—H01	109.5
C26—C25—H25	120.1	C11—P1—C21	105.79 (9)
C24—C25—H25	120.1	C11—P1—C31	104.93 (9)
C25—C26—C21	120.2 (2)	C21—P1—C31	104.07 (9)
C25—C26—H26	119.9	C11—P1—Ir1	114.58 (7)
C21—C26—H26	119.9	C21—P1—Ir1	113.22 (7)
C36—C31—C32	119.51 (19)	C31—P1—Ir1	113.28 (7)
C36—C31—P1	120.43 (16)	C41—P2—C51	105.55 (10)
C32—C31—P1	119.92 (16)	C41—P2—C61	104.08 (9)
C33—C32—C31	119.8 (2)	C51—P2—C61	106.46 (9)
C33—C32—H32	120.1	C41—P2—Ir1	114.40 (7)
C31—C32—H32	120.1	C51—P2—Ir1	110.59 (7)
C34—C33—C32	120.2 (2)	C61—P2—Ir1	114.98 (7)
C34—C33—H33	119.9	F4—P3—F1	91.02 (14)
C32—C33—H33	119.9	F4—P3—F2	178.61 (15)
C33—C34—C35	120.4 (2)	F1—P3—F2	90.22 (14)
C33—C34—H34	119.8	F4—P3—F5	92.35 (13)
C35—C34—H34	119.8	F1—P3—F5	91.35 (11)
C36—C35—C34	119.7 (2)	F2—P3—F5	88.26 (12)
C36—C35—H35	120.2	F4—P3—F3	89.76 (12)
C34—C35—H35	120.2	F1—P3—F3	179.21 (13)
C35—C36—C31	120.4 (2)	F2—P3—F3	88.99 (12)
C35—C36—H36	119.8	F5—P3—F3	88.74 (9)
C31—C36—H36	119.8	F4—P3—F6	89.48 (12)
C42—C41—C46	119.6 (2)	F1—P3—F6	89.89 (11)
C42—C41—P2	119.11 (16)	F2—P3—F6	89.89 (10)
C46—C41—P2	120.93 (16)	F5—P3—F6	177.78 (11)
C43—C42—C41	120.3 (2)	F3—P3—F6	90.00 (9)
C43—C42—H42	119.9	C2—Ir1—C1	126.42 (10)
C41—C42—H42	119.9	C2—Ir1—C3	115.45 (9)
C44—C43—C42	119.8 (2)	C1—Ir1—C3	118.13 (9)
C44—C43—H43	120.1	C2—Ir1—P2	88.65 (6)
C42—C43—H43	120.1	C1—Ir1—P2	90.26 (6)
C43—C44—C45	120.4 (2)	C3—Ir1—P2	90.49 (6)
C43—C44—H44	119.8	C2—Ir1—P1	89.28 (6)
C45—C44—H44	119.8	C1—Ir1—P1	89.27 (6)
C44—C45—C46	120.0 (2)	C3—Ir1—P1	92.30 (6)
C44—C45—H45	120	P2—Ir1—P1	177.047 (18)
C46—C45—H45	120		
			
C16—C11—C12—C13	0.1 (3)	C12—C11—P1—C31	26.42 (19)
P1—C11—C12—C13	176.57 (16)	C16—C11—P1—Ir1	−32.28 (19)
C11—C12—C13—C14	−0.3 (3)	C12—C11—P1—Ir1	151.30 (14)
C12—C13—C14—C15	0.1 (3)	C22—C21—P1—C11	2.27 (19)
C13—C14—C15—C16	0.3 (3)	C26—C21—P1—C11	177.92 (16)
C12—C11—C16—C15	0.3 (3)	C22—C21—P1—C31	−108.02 (17)
P1—C11—C16—C15	−176.18 (17)	C26—C21—P1—C31	67.63 (17)
C14—C15—C16—C11	−0.5 (3)	C22—C21—P1—Ir1	128.54 (15)
C26—C21—C22—C23	−1.1 (3)	C26—C21—P1—Ir1	−55.80 (17)
P1—C21—C22—C23	174.49 (16)	C36—C31—P1—C11	91.01 (18)
C21—C22—C23—C24	−0.2 (3)	C32—C31—P1—C11	−84.74 (18)
C22—C23—C24—C25	1.0 (3)	C36—C31—P1—C21	−158.07 (17)
C23—C24—C25—C26	−0.5 (3)	C32—C31—P1—C21	26.19 (19)
C24—C25—C26—C21	−0.9 (3)	C36—C31—P1—Ir1	−34.68 (18)
C22—C21—C26—C25	1.7 (3)	C32—C31—P1—Ir1	149.57 (14)
P1—C21—C26—C25	−174.07 (16)	C42—C41—P2—C51	161.74 (16)
C36—C31—C32—C33	−0.6 (3)	C46—C41—P2—C51	−24.91 (19)
P1—C31—C32—C33	175.17 (16)	C42—C41—P2—C61	−86.35 (18)
C31—C32—C33—C34	0.4 (3)	C46—C41—P2—C61	86.99 (18)
C32—C33—C34—C35	0.2 (4)	C42—C41—P2—Ir1	39.93 (18)
C33—C34—C35—C36	−0.6 (4)	C46—C41—P2—Ir1	−146.72 (15)
C34—C35—C36—C31	0.4 (3)	C56—C51—P2—C41	124.9 (2)
C32—C31—C36—C35	0.2 (3)	C52—C51—P2—C41	−58.94 (19)
P1—C31—C36—C35	−175.57 (17)	C56—C51—P2—C61	14.6 (2)
C46—C41—C42—C43	−0.7 (3)	C52—C51—P2—C61	−169.15 (17)
P2—C41—C42—C43	172.75 (17)	C56—C51—P2—Ir1	−110.90 (19)
C41—C42—C43—C44	0.1 (3)	C52—C51—P2—Ir1	65.30 (18)
C42—C43—C44—C45	0.9 (4)	C66—C61—P2—C41	151.24 (17)
C43—C44—C45—C46	−1.2 (4)	C62—C61—P2—C41	−29.95 (19)
C44—C45—C46—C41	0.6 (3)	C66—C61—P2—C51	−97.51 (18)
C42—C41—C46—C45	0.3 (3)	C62—C61—P2—C51	81.29 (18)
P2—C41—C46—C45	−173.00 (17)	C66—C61—P2—Ir1	25.32 (19)
C56—C51—C52—C53	−1.5 (3)	C62—C61—P2—Ir1	−155.87 (15)
P2—C51—C52—C53	−177.75 (18)	C41—P2—Ir1—C2	154.66 (10)
C51—C52—C53—C54	−0.3 (4)	C51—P2—Ir1—C2	35.65 (10)
C52—C53—C54—C55	1.9 (4)	C61—P2—Ir1—C2	−84.94 (10)
C53—C54—C55—C56	−1.7 (4)	C41—P2—Ir1—C1	−78.91 (10)
C52—C51—C56—C55	1.6 (4)	C51—P2—Ir1—C1	162.08 (10)
P2—C51—C56—C55	177.8 (2)	C61—P2—Ir1—C1	41.48 (10)
C54—C55—C56—C51	0.0 (4)	C41—P2—Ir1—C3	39.22 (10)
C66—C61—C62—C63	−0.1 (3)	C51—P2—Ir1—C3	−79.79 (10)
P2—C61—C62—C63	−178.91 (17)	C61—P2—Ir1—C3	159.61 (9)
C61—C62—C63—C64	−0.3 (3)	C11—P1—Ir1—C2	−156.99 (10)
C62—C63—C64—C65	0.3 (4)	C21—P1—Ir1—C2	81.56 (10)
C63—C64—C65—C66	0.3 (4)	C31—P1—Ir1—C2	−36.64 (10)
C62—C61—C66—C65	0.6 (3)	C11—P1—Ir1—C1	76.58 (10)
P2—C61—C66—C65	179.41 (17)	C21—P1—Ir1—C1	−44.87 (10)
C64—C65—C66—C61	−0.7 (3)	C31—P1—Ir1—C1	−163.08 (10)
C16—C11—P1—C21	93.15 (18)	C11—P1—Ir1—C3	−41.55 (9)
C12—C11—P1—C21	−83.26 (18)	C21—P1—Ir1—C3	−163.00 (9)
C16—C11—P1—C31	−157.16 (17)	C31—P1—Ir1—C3	78.80 (10)

### Hydrogen-bond geometry (Å, º)

Cg1, Cg2 and Cg3 are the centroids of the C11–C16, C21–C26 and C41–C46 rings, respectively.

**Table:**

*D*—H···*A*	*D*—H	H···*A*	*D*···*A*	*D*—H···*A*
C15—H15···F3^i^	0.95	2.39	3.281 (3)	157
C16—H16···F6^i^	0.95	2.53	3.319 (3)	141
C42—H42···F6^i^	0.95	2.38	3.138 (3)	136
C43—H43···F2^i^	0.95	2.49	3.386 (3)	158
C45—H45···O01^ii^	0.95	2.50	3.281 (3)	139
C64—H64···F4^iii^	0.95	2.47	3.200 (3)	133
O01—H01···F3^i^	0.84	2.27	3.059 (3)	157
C53—H53···*Cg*1^iv^	0.95	2.68	3.523 (2)	148
C35—H35···*Cg*3^iv^	0.95	2.91	3.587 (2)	129
C13—H13···*Cg*2^v^	0.95	2.97	3.744 (2)	140

Symmetry codes: (i) *x*, −*y*+3/2, *z*+1/2; (ii) −*x*+1, *y*+1/2, −*z*+3/2; (iii) −*x*+2, −*y*+2, −*z*+1; (iv) −*x*+1, −*y*+1, −*z*+1; (v) −*x*+2, *y*−1/2, −*z*+3/2.
